# Associations Between Health Literacy, Environmental Factors, and Fall Prevention Behaviors Among Community-Dwelling Older Adults in Northern Thailand: A Cross-Sectional Study

**DOI:** 10.3390/ijerph23030281

**Published:** 2026-02-25

**Authors:** Boonsita Suwannakul, Arunrat Srithawong, Chonticha Kaewjoho, Thapakorn Ruanjai, Chatchada Sutalangka, Ploypailin Namkorn, Ekalak Sitthipornvorakul, Siripatra Atsawakaewmongkhon, Raksuda Taniguchi, Aunyachulee Ganogpichayagrai, Wilawan Chaiut

**Affiliations:** 1 Department of Physical Therapy, Faculty of Allied Health Sciences, University of Phayao, Phayao 56000, Thailand; boonsita.su@up.ac.th (B.S.); arunrat.sr@up.ac.th (A.S.); chonticha.ka@up.ac.th (C.K.); 2 Department of Public health, School of Health Science, Mae Fah Luang University, Chiang Rai 57100, Thailand; thapakorn.rua@mfu.ac.th; 3 Department of Physical Therapy, School of Integrative Medicine, Mae Fah Luang University, Chiang Rai 57100, Thailand; chatchada.sut@mfu.ac.th (C.S.); ploypailin.nam@mfu.ac.th (P.N.); ekalak.sit@mfu.ac.th (E.S.); siripatra.ats@mfu.ac.th (S.A.); 4 Department of Traditional Chinese Medicine, School of Integrative Medicine, Mae Fah Luang University, Chiang Rai 57100, Thailand; raksuda.kle@mfu.ac.th; 5 Department of Applied Thai Traditional Medicine, School of Integrative Medicine, Mae Fah Luang University, Chiang Rai 57100, Thailand; aunyachulee.gan@mfu.ac.th

**Keywords:** health information, aged, accidental falls, health behavior, home environment

## Abstract

**Highlights:**

**Public health relevance—How does this work relate to a public health issue?**

- Falls among community-dwelling older adults are a major public health concern, and limited health literacy is a modifiable risk factor.
- This study links health literacy with home environment features and fall prevention behaviors in an Asian community setting, where evidence is scarce.

**Public health significance—Why is this work of significance to public health?**

- More than half of older adults exhibited limited health literacy, highlighting a substantial population-level vulnerability.
- Higher health literacy was associated with key protective factors, including regular exercise, safer home sanitation (sitting toilets), and proper footwear.

**Public health implications—What are the key implications or messages for practitioners, policy makers and/or researchers?**

- Fall prevention programs should integrate health literacy improvement with exercise promotion, home safety modifications, and safe footwear education.
- Policies and community interventions targeting older adults should address both individual health literacy and environmental safety to reduce fall risk.

**Abstract:**

Inadequate health literacy (HL) is a critical factor contributing to fall risk among older adults. However, evidence on how HL relates to home environment and fall prevention behaviors in Asian community settings remains limited. This study aimed to assess HL levels and examine their associations with sociodemographic characteristics, home environment, and fall prevention behaviors. A community-based cross-sectional study was conducted among 177 community-dwelling older adults aged 60 to 79 years. Data were collected through structured questionnaires assessing sociodemographic characteristics, home environment, fall prevention behaviors, and HL using the European Health Literacy Survey Questionnaire (HLS-EU-Q47), which cover three subdomains: healthcare, disease prevention, and health promotion. Multiple linear regression analysis was performed to examine the associations between sociodemographic factors, home environment, fall prevention behaviors, and HL. The HL score among older adults was 34.50 ± 7.50 (54.2% limited HL). A total HL score was positively associated with regular exercise (β = 2.73, 95% CI: 0.71, 4.74) and a sitting toilet (β = 6.38; 95% CI: 3.83, 8.92) and marginally associated with wearing properly fitting shoes (β = 2.54; 95% CI: 0.22, 4.86). Therefore, the health promotion aimed at improving HL in this population may benefit from concurrently promoting regular exercise, home safety modifications, and safe footwear practices.

## 1. Introduction

Aging is often accompanied by physical and cognitive declines, resulting in multiple health-related challenges [1,2]. Additionally, falls among older adults have broad social and economic implications. The prevalence of falls among community-dwelling older adults has been reported as 8.2% in Europe [2]. In Asia, prevalence estimates reach 16.5%, with an annual incidence rate of approximately 31% [3,4]. Beyond causing physical injuries, falls are a leading cause of fractures—particularly hip fractures—which significantly increase healthcare costs [5]. The causes of falls are multifactorial, encompassing both intrinsic and extrinsic factors. Intrinsic risk factors include age-related physiological changes, chronic conditions, balance impairment, medication use, and behavioral elements such as HL, whereas extrinsic risk factors primarily involve environmental hazards such as poor lighting and slippery floors [6,7]. Evidence from Japan indicates that HL is significantly associated with gait speed among community-dwelling older adults, suggesting that HL may influence physical functioning, given that gait speed reflects frailty and disease status [8]. 

HL refers to an individual’s ability to acquire, understand, and use the health information and services necessary for making informed health-related decisions [9,10]. HL enables individuals to engage effectively with health services, make informed lifestyle choices, and participate in disease prevention and health promotion activities [11]. The European Health Literacy Survey Questionnaire (HLS-EU-Q47) provides a multidimensional framework to assess HL across healthcare, disease prevention, and health promotion domains through the dimensions of access, understanding, appraisal, and application [12]. Previous studies have reported that lower HL levels are correlated with a higher risk of falls among hospitalized patients [13,14]. Conversely, higher HL levels are associated with greater participation in fall prevention activities, such as physical exercise and balance training, as well as lower fear of falling [15]. 

Existing evidence indicates that HL is associated with various health-related behaviors, including those linked to fall prevention among older adults. A prior survey of hospitalized elderly patients demonstrated a significant association between HL and fall prevention behaviors, highlighting the potential for HL to influence safety-related actions. However, most older adults reside not in institutional care facilities but in community settings, where access to medical information and support is often constrained [16,17,18]. Li et al. [19] reported that adequate HL was related to a lower prevalence of falls among older adults; however, this relationship was only significant among men. Similarly, research in Korea found that HL levels were associated with residential areas and participation in daily activities in adults aged 65 years and above. Despite these findings, empirical evidence examining the relationships among HL, home environment, and specific fall prevention behaviors in community-dwelling Asian populations remains scarce [20]. Previous studies have examined HL among general populations and older adults, including those in Thailand [16,17]. However, research focusing specifically on community-dwelling older adults remains limited.

This study is guided by the perspective that health behaviors are influenced by both individual and environmental determinants. HL represents an individual capacity that enables older adults to access, understand, and apply information related to fall prevention. Those with higher health literacy may be better able to recognize fall risks and adopt preventive behaviors. At the same time, home environmental conditions constitute important contextual factors that may either increase fall risk or support prevention. Therefore, this study examines how health literacy, sociodemographic characteristics, and home environmental factors are associated with fall-prevention behaviors among community-dwelling older adults.

## 2. Materials and Methods

### 2.1. Study Design and Setting

This cross-sectional survey was conducted from January to March 2025 to assess HL levels among older adults and identify key factors across three subdomains: healthcare, disease prevention, and health promotion. The study took place in a community setting in Phayao Province, Northern Thailand. The study was approved by the Human Ethics Committee at the University of Phayao (protocol code HREC-UP-HSST 1.2/015/68).

### 2.2. Study Population

The inclusion criteria were as follows: (1) aged 60–79 years; (2) independent in activities of daily living (ADL), as determined through a brief researcher-administered assessment using the Barthel ADL Index (score > 12, total score = 20) [21]; and (3) proficient in understanding and speaking Thai. The participants were recruited via convenience sampling. Recruitment posters were displayed at community centers, temples, and public markets within Phukamyao District. The study site represented a typical semi-urban area in Northern Thailand with a high proportion of community-dwelling older adults and established local public health networks, making it an appropriate and representative context for examining HL and fall prevention behaviors. Additionally, community health volunteers personally approached eligible older adults to provide study information. Those interested were invited to attend an information session where eligibility screening and informed consent procedures were conducted. 

### 2.3. Study Sample and Sample Size Calculation

The sample size was calculated using G*Power version 3.1.9.7. The effect size was derived from a previous study reporting a significant negative correlation between total HL scores and fall behavior scores (r = −0.218, *p* < 0.01) [17]. According to Cohen’s conventions, this corresponds to a small-to-medium effect size (r^2^ = 0.048; f^2^ = 0.05). Using this effect size (f^2^ = 0.05), an alpha level of 0.05, and a desired power of 0.80, the minimum required sample size was 177 participants. 

### 2.4. Research Instruments

The participants were interviewed about sociodemographic characteristics, home environmental factors, and fall prevention behaviors. HL was measured using the Thai version of the 47-item instrument HLS-EU-Q47. Sociodemographic factors included age (years), sex, marital status, education level (elementary, secondary, or bachelor’s degree or higher), comorbidity (yes or no), number of medications (none, fewer than 4, or 4 or more types), cigarette smoking, alcohol consumption, regular exercise (3–5 days or more than 150 min per week) (yes or no), village health volunteer status (yes or no), living with family (yes or no), history of falls in the past 6 months, gait aid use (yes, using a cane, walker, or crutch for community walking, or no), vision (clear, clear with glasses, or not clear), and hearing (clear, clear with hearing aids, or not clear). Village health volunteer status was defined as whether the participant was currently registered and actively serving as a village health volunteer under the Thai Ministry of Public Health (yes or no). Village health volunteers are trained community members who assist in health promotion, disease prevention, and basic public health services at the community level. A fall was defined as an unexpected event in which the participant comes to rest on the ground, floor, or a lower level [22]. Participants were informed of this definition at enrollment. Slips and trips were classified as falls only if they resulted in contact with the ground or a lower surface. Near misses without ground contact were not classified as falls. Falls were assessed using a six-month recall period. This timeframe was selected to balance recall accuracy and adequate event capture, as longer recall periods are associated with increased recall bias and underreporting [23]. It should be noted that the “not clear and have no glasses/hearing aids” category conflates sensory impairment with a potential lack of access to corrective devices, which is a socioeconomic factor. Exercise was dichotomized based on World Health Organization (WHO) recommendations [24] for analytical purposes, acknowledging that this may obscure dose–response relationships. Gait aid use and living with family were also coded as binary variables. 

Participants then answered “yes” or “no” questions about home environmental factors and fall prevention behaviors. The home environment questionnaire assessed whether each participant’s house had a ground-floor bedroom, level and dry floors, handrails on stairs, clear walkways, sufficient lighting, handrails in the bathroom, a sitting toilet, an elevated bed, a kitchen countertop, and even surfaces around the house. The home environment checklist and fall prevention behavior items were based on previously validated instruments used in community-dwelling older adults with an index of item objective congruence (IOC) value of 0.80 for all items [25]. 

The fall prevention behaviors questionnaire evaluated the participants’ usual practices to prevent falls, including wearing properly fitting shoes, turning on the bathroom light, wiping water spilled on the floor, sitting while showering, changing positions slowly, wearing appropriately sized clothing, cleaning the house, using handrails, using a walking aid if advised, and undergoing annual fall risk screening. In Thailand, annual fall risk screening is included in routine preventive services offered through primary care networks, including subdistrict health promotion hospitals and community health volunteers. These screenings are typically conducted by trained health personnel or village health volunteers using simple questionnaires and functional assessments during wellness visits or community outreach events. The fall prevention behaviors questionnaire was developed based on evidence-based fall prevention guidelines and previous empirical studies identifying modifiable behavioral risk factors among community-dwelling older adults. The final instrument included 10 items. The questionnaire contents were examined by three experts from the areas of gerontological nursing, mental health, and public health and were revised before a pilot study was performed on 30 older adults. The Cronbach’s alpha coefficient reported the reliability of the questionnaire as 0.78.

The European Health Literacy Survey Questionnaire (HLS-EU-Q47) scoring system is used to assess an individual’s health literacy level based on their responses to 47 questions. The conceptual model of the HLS-EU-Q47 distinguishes between three contexts of HL (health care, disease prevention, and health promotion) and four competencies to deal with information relative to health (access, understand, appraise, and apply). The scoring method involves converting responses into a numerical scale and categorizing individuals into different levels of health literacy. Each item in the questionnaire is rated on a 4-point Likert scale including very difficult (1), difficult (2), easy (3), and very easy (4) [10]. To standardize HL scores on a scale from 0 to 50, the following formula is applied:

HL Index = (mean − 1) × (50/3)(1)

In this formula, the “Mean Score” represents the average response in each index. Based on the calculated HL index score, the HLS-EU-Q47 defines four health literacy levels including inadequate HL (0–25 scores), problematic HL (>25−33 scores), sufficient HL (>33–42 scores), and excellent HL (>42–50 scores). For identifying vulnerable groups, the “inadequate” and “problematic” HL categories were combined into a single group termed “limited HL,” encompassing scores from 0 to 33. We used the Thai version of the HLS-EU-Q47, which was previously translated and psychometrically validated in Thai population [26]. The validated version demonstrated good internal consistency (Cronbach’s α = 0.88). The index of item objective congruence (IOC) score was >0.5.

All the volunteers were interviewed by researchers who were thoroughly trained in administering the questionnaires.

### 2.5. Statistical Analysis

The collected data were analyzed using Stata/SE version 17 (StataCorp LLC, College Station, TX, USA). Descriptive statistics, including means and standard deviations (SD) for continuous variables and frequencies (n) and percentages (%) for categorical variables, were calculated to summarize sociodemographic characteristics, home environmental factors, and fall prevention activities among older adults. All binary variables were coded numerically, with “yes” responses assigned a value of 1 and “no” responses assigned a value of 0. Multiple linear regression analysis was employed to investigate factors associated with HL scores. Before the regression model was developed, standardized residuals for the dependent variables and multicollinearity for the independent variables were investigated. To assess the presence of multicollinearity, the variance inflation factor (>5) for all independent variables in the regression model was calculated. The findings indicated that the model had no multicollinearity, with the greatest variance inflation factor being 3.47. Potential outliers were assessed using standardized residuals and Cook’s distance. No cases exceeded the recommended cut-offs (standardized residuals > ±3; Cook’s distance > 1). Therefore, all the data were retained for analysis. Interaction terms between independent variables were not tested due to the limited sample size and the primary theoretical focus on main-effect associations. Including interaction terms in this dataset would have increased the risk of model instability and overfitting.

Variables were selected for inclusion in the multiple linear regression model based on prior theoretical considerations and empirical evidence. Although univariable analyses (independent *t*-test and one-way ANOVA) were conducted to explore associations with HL scores, all theoretically relevant variables were retained in the multivariable regression model regardless of their univariable statistical significance. The variables considered included sociodemographic characteristics (monthly income, comorbidities, cigarette smoking, regular exercise, living with family, and use of gait aids), home environmental factors (bathroom handrails, sitting toilets, kitchen countertops), and fall prevention behaviors (wearing properly fitting shoes and turning on bathroom lights). Assumptions of residual normality and homoscedasticity were verified using both visual and numerical tests. Residual normality was examined using a histogram and Q–Q plots of standardized residuals and confirmed with the Shapiro–Wilk test (*p* > 0.05). Homoscedasticity was assessed through scatter plots of standardized residuals versus predicted values and Levene’s test for equality of variances, indicating constant variance across fitted values.

## 3. Results

### 3.1. Descriptive Statistics of Sociodemographic Characteristics, Environmental Factors, and Fall Prevention Behaviors Among Older Adults

Of the 177 volunteers, the average age of participants was 68.3 ± 5.9 years, and 70.1% were female. About 66% were married, 84% had an elementary school education or less, and 92% reported a monthly income of ≤150 USD. Approximately 23% of the participants served as village health volunteers. A total of 52.5% had comorbidities, such as controlled hypertension, type 2 diabetes, or dyslipidemia, with 15.8% taking four or more medications. Around 6.8% smoked cigarettes, while 26% drank alcohol. A small percentage (15.3%) experienced a fall in the past six months, and 4% used gait aids. Nearly all the participants (85.3%) reported normal hearing, and 46.3% had normal vision (Table 1). 

Home environmental factors reported by over 80% of participants included level and dry floors, handrails on stairs, clear walkways, sufficient lighting, platform beds raised to knee level, kitchen countertops, and smooth surfaces throughout the house. Regarding fall prevention behaviors, more than 80% of the participants reported consistently turning on bathroom lights, wiping water spilled on the floor, wearing properly fitting clothing, and keeping their homes clean (Table 2).

### 3.2. Health Literacy Among Older Adults

**Figure 1:**
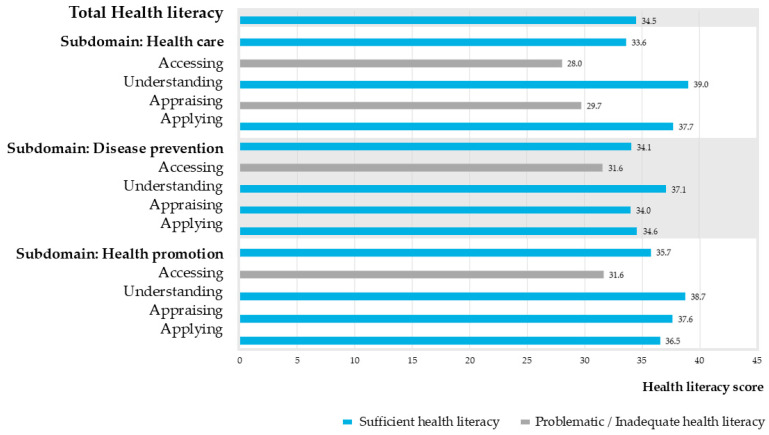
Mean scores for each subdomain of health literacy among older adults (n = 177). Bar colors indicate whether the mean value exceeds the sufficiency threshold: blue bar represent score ≥ 33; sufficient HL and gray bar represent score < 33; inadequate HL.

The results showed that 81 participants (45.8%) demonstrated sufficient HL (score > 33), while 96 participants (54.2%) exhibited limited HL (inadequate or problematic HL, score 0–33). However, the distribution is skewed, with a minority having very low scores pulling the average into the sufficient range. The average total HL score among the older adults was 34.5 ± 7.5, indicating an overall sufficient HL level. Analysis by domain revealed mean scores of 33.6 for the healthcare subdomain, 34.1 for disease prevention, and 35.7 for health promotion. All three subdomains were categorized as sufficient HL. However, some subdomain components had mean scores below 34, indicating inadequate HL levels. These included the accessing and appraising components of the healthcare subdomain (28.0 ± 11.6 and 29.7 ± 11.2, respectively), the accessing component of the disease prevention subdomain (31.6 ± 11.8), and the appraising component of the health promotion subdomain (31.6 ± 11.4) (Figure 1).

### 3.3. Univariable Analysis Identified Factors Associated with Health Literacy Scores Among Older Adults

The univariable analysis identified several factors significantly associated with HL among older adults across the total score (Table A1 in Appendix A). The analysis of sociodemographic parameters indicated that older adults with a higher monthly income (>150 USD/month) scored higher in HL compared to those with lower incomes (*p* = 0.045). In addition, older adults who did not have co-morbidity demonstrated greater HL than those who had some co-morbidity (0.010). Older adults who lived with family scored significantly higher than those who lived alone (*p* = 0.036). Furthermore, older adults who engaged in regular exercise achieved higher HL scores across compared to those with non-regular or non-exercise (*p* = 0.006). Older adults who reported no smoking exhibited higher HL scores compared to those with smoking (*p* = 0.038). Regarding gait aid use, older adults who reported no gait aid use scored higher in HL compared to those who applied (*p* = 0.036). The analysis of home environmental factors indicated that older adults whose house with handrails in the bathroom, sitting toilet, and countertop kitchen demonstrated greater HL than those whose house without these (*p* = 0.006, *p* < 0.001, and *p* < 0.001, respectively). Regarding fall prevention behaviors, older adults who usually wear properly fitting shoes and turning-on the light in the bathroom scored significantly higher than those who were not (*p* < 0.001 and *p* = 0.004, respectively). 

### 3.4. Multivariate Results Identified Factors Associated with HL Scores Among Older Adults

The multivariable analysis identified significant factors associated with HL among older adults across the total score and within the three HL subdomains (Table 3). Among sociodemographic factors, regular exercise was significantly positively associated with the total HL score as well as with the healthcare and disease prevention subdomains. Conversely, gait aid use was negatively associated with the healthcare and health promotion subdomains. Regarding home environmental factors, having a sitting toilet was positively associated with the total HL score and the healthcare and disease prevention subdomains. Concerning fall prevention behavior factors, wearing properly fitting shoes was positively associated with the total HL score.

Of the 81 participants with good health literacy, 24 (29.6%) were village health volunteers. Overall, 24 of 41 village health volunteers (58.5%) had good health literacy compared with 57 of 136 non-village health volunteers (41.9%). However, in univariable analysis, village health volunteer status was not significantly associated with health literacy scores (*p* = 0.109), and therefore it was not included in the multivariable linear regression model. To determine whether village health volunteer status acted as a true confounder, we conducted a sensitivity analysis excluding village health volunteers (n = 41). The results of the multiple linear regression were materially unchanged, with the same predictors remaining statistically significant and effect estimates showing minimal variation. These findings suggest that village health volunteer status did not meaningfully confound the associations observed in this study.

## 4. Discussion

In this study, we assessed the HL levels of 177 community-dwelling older adults aged 60 years and above and examined their associations with sociodemographic characteristics, home environment, and fall prevention behaviors. The findings indicated that the average total HL score among the older adults was within sufficient range. However, limited or inadequate HL was observed in the accessing and appraising components of the healthcare subdomain, the accessing component of the disease prevention subdomain, and the appraising component of the health promotion subdomain. 

Sociodemographic factors, particularly regular exercise and the use of gait aids, showed significant associations with HL levels among community-dwelling older adults. A positive association was observed between regular exercise and overall HL scores, including the healthcare and disease prevention subdomains. Older adults who engage in regular physical activity may also demonstrate better cognitive function [27], and prior research has indicated that cognitive ability is related to HL, particularly in processing and understanding health information [28]. Regular exercise may enhance appraisal skills by increasing exposure to health information, peer interactions, and health-promoting routines that reinforce decision-making. Similarly, social support from the family may facilitate navigation and access to reliable health information.

Beyond potential cognitive benefits, regular exercise among Thai older adults often occurs in social settings, such as community exercise groups or senior clubs. Participation in such activities provides opportunities for social interaction, peer learning, and the exchange of health-related information. These experiments may increase exposure to credible health messages and foster mutual support, which could correspond with higher HL levels. Our results are consistent with previous studies that have identified associations between regular physical activity and HL among older adults [29]. Fernandez et al. [30] reported that older adults with sufficient HL were more likely to participate in moderate exercise two or more times per week than those with lower HL. Conversely, unhealthy lifestyle habits—such as physical inactivity, poor diet, and infrequent medical check-ups—have been associated with lower HL levels [31]. 

Using gait aids was negatively associated with HL scores in the healthcare and health promotion subdomains. The wide confidence intervals observed for some predictors were due to the small subgroup size. One possible explanation is that the use of gait aids may correspond with reduced opportunities for physical and social engagement, thereby limiting exposure to health information [32,33]. However, this explanation remains speculative and requires further research. Gait speed has been associated with both physical and cognitive functions, as well as attention, which are key factors for reading comprehension and closely linked to HL [34,35]. In addition, previous studies have shown positive correlations between gait speed and HL among community-dwelling older adults [8,34]. Using gait aids might also relate to decreased social participation, as shown by Kawakami et al., who reported a higher hazard ratio for disability among individuals with both low HL and low social activity [35]. Individuals requiring gait aids may have higher levels of physical frailty, which has been associated in prior studies with reduced cognitive reserve, limited health information processing, and lower participation in health-promoting activities.

Interestingly, traditional sociodemographic variables—such as age, sex, and educational attainment—were not significant predictors of HL in this study. This pattern may reflect the relative homogeneity of the sample, in which most participants had low educational attainment (84% with elementary education or less) and a low monthly income (92%). When key characteristics show limited variability, their statistical associations with HL are naturally constrained. Chiu et al. [36] and Lima et al. [37] similarly identified lower education, cognitive decline, limited socioeconomic resources, chronic illness, and restricted access to health information to be key correlates of poor HL. The relatively young age profile of our sample may have influenced the associated risk factors. As fall risk increases with age due to sarcopenia, balance impairment, multimorbidity, and polypharmacy [38]. Although this homogeneity limits the generalizability of our findings to more socioeconomically diverse populations, it strengthens our ability to examine other contextual and behavioral factors that may influence HL within this specific demographic group [39]. Future studies involving more diverse samples are needed to clarify whether these variables retain their predictive value across different socioeconomic settings. Village health volunteers might be expected to have higher health literacy due to their training and involvement in community health activities. However, although village health volunteers receive basic health training, village health volunteer status was not significantly associated with HL scores in our sample. In addition, sensitivity analysis excluding village health volunteers yielded similar results, indicating that village health volunteer status did not materially influence the observed associations. These suggest that participation in the village health volunteers program alone may not necessarily translate into higher overall HL, or that HL levels among village health volunteers and the broader community were comparable.

The home environment was also related to HL levels. The presence of a sitting toilet was positively associated with overall HL scores and with the healthcare and disease prevention subdomains. This relationship may reflect underlying differences in socioeconomic status (SES), health awareness, or access to information among those living in homes with safer facilities, such as sitting toilets. A sitting toilet can support hygiene practices and reduce risks related to squatting or balance instability [39,40,41,42]. Prior studies have shown that a safer home environment, including bathroom modifications, is associated with a lower risk of falls [19,40]. However, it is equally plausible that individuals with higher HL—or those with greater resources and health awareness—are more likely to modify their home environments to include safety features such as sitting toilets. Although our study found that having a sitting toilet was associated with a 6.38-point difference in HL scores, this finding should be interpreted as exploratory and hypothesis-generating. While the effect size is noteworthy, its practical significance remains uncertain. Future studies using larger sample sizes and controlling potential confounding factors are needed to clarify these associations. In addition, the sitting toilet variable is likely a proxy indicator for a higher SES and environmental context. Future research needs to disentangle these relationships by directly measuring SES, wealth, and urbanization.

Regarding fall prevention behaviors, we observed a significant positive association between wearing properly fitting shoes and overall HL scores. Notably, approximately one-quarter of participants reported wearing ill-fitting footwear, such as slippers or flip-flops, outside the home, which may increase fall risk. This finding aligns with previous research demonstrating that higher levels of health literacy are associated with safer health-related behaviors. For example, negative correlations have been reported between total scores on the Falls Behavioral Scale and the understanding and appraisal subdimensions of the HLS-EU-Q47 [20], suggesting that limited health literacy may reduce engagement in protective behaviors. Additionally, other studies have shown that the adoption of fall prevention practices is associated with proactive health behaviors, such as regular self-assessment of health status, as well as higher education levels [14]. Many older adults do not regularly inspect their feet, are unaware of their correct shoe size, or have trouble selecting footwear that accommodates variations in foot shape [43]. Footwear is an important factor related to fall prevention, as it contributes to stability and protection against slips, trips, and falls (STFs)—common accidents that often result in injuries among older adults [44]. Key safety features include proper fit, secure fastening, appropriate heel and collar height, slip resistance, and suitable sole or insole hardness [35]. Proper fitting shoes are important, as both loose and tight footwear have been associated with instability and discomfort. One study reported a significant difference in fall occurrence within the past six months between community-dwelling older adults who wore ill-fitting shoes (56%) and those who wore properly fitting shoes [43]. Similarly, Kim and Hegazy [44] highlighted that appropriate footwear design—such as slip-resistant soles, correct heel height, cushioning, and secure fastening—was related to a lower risk of falls. In addition, a comparative observational study indicated that older adults with well-fitting shoes tended to demonstrate better balance and a lower fear of falling than those wearing ill-fitting shoes [45]. The ability to appraise footwear risks and understand the benefits of properly fitting shoes may reflect aspects of functional HL. However, further research is needed to clarify whether higher HL promotes better footwear choices or whether individuals who already practice safety behaviors develop higher HL through greater engagement with health information.

The presence of annual fall risk screening within Thailand’s primary care system may have influenced our findings in several ways. Participants who regularly attend preventive health services may have previously received fall risk assessment, education, or referral for preventive interventions (e.g., exercise programs or home safety advice), potentially reducing their risk of falls. Conversely, individuals who are socially isolated or have mobility limitations may be less likely to participate in routine screening and therefore may remain at higher risk. The voluntary and variable coverage of the program may contribute to heterogeneity in fall risk and preventive exposure within our sample. However, given the inconsistent follow-up and uncertain population-level reach of the screening program, its overall impact on our study outcomes is likely limited.

The observed associations between HL and fall prevention behaviors can be interpreted through behavioral change frameworks such as the health belief model (HBM) and social cognitive theory (SCT). Within these frameworks, HL relates to key constructs such as knowledge acquisition, perceived risk, perceived benefits, and self-efficacy. Older adults with higher HL may be better equipped to interpret health information and apply it to preventive actions such as regular exercise or appropriate footwear use. In addition, social and community contexts that support HL—such as group exercise and health education programs—may reinforce motivation and self-regulation, contributing to sustained engagement in health-protective behaviors [46]. The integration of HBM and SCT provides a useful foundation for interpreting these findings and guiding future intervention design. Within HBM, regular exercise and safe home behaviors may strengthen perceived benefits and reduce perceived susceptibility to falls, thereby promoting protective actions. From the SCT perspective, self-efficacy plays a central role, and our results suggest that interventions aiming to enhance confidence—such as supervised group exercise sessions, peer-led demonstrations of safe mobility techniques, or modeling appropriate fall prevention behaviors—may be particularly beneficial for older adults with limited HL. Strategies such as providing verbal encouragement, fostering social support within community networks, and enabling opportunities for mastery experiences could further reinforce fall prevention behaviors. Thus, the findings highlight how theoretically grounded approaches could be translated into practical, community-based programs tailored for older adults in similar semi-urban Thai settings.

This study has several limitations. First, the use of convenience sampling introduces potential selection bias, limiting the ability to generalize to all community-dwelling older adults, especially those who are socially isolated, less mobile, or have low health motivation. Future research should apply systematic methods, such as random sampling from community registries, to improve representativeness. Second, as this research was conducted in a single community in Northern Thailand, regional differences may limit external validity. Third, behavioral and home environmental data were self-reported and therefore subject to recall and social desirability biases. Fourth, certain variables (e.g., exercise, gait aid use, vision, hearing, and properly fitting shoes) were collected in binary form, potentially masking gradations in these characteristics. The “not clear and have no glasses/aids” categories combine biological and socioeconomic elements, which should be distinguished in future research. In addition, future studies should consider continuous variables (e.g., duration of exercise in minutes, MET-minutes/week) or more detailed ordinal scales to capture greater variability and enhance analytical precision. Finally, wide confidence intervals indicate small subgroup sizes (for example, gait aid use in the health promotion model: β = −7.48, 95% CI: −14.47, −0.49), and some nonsignificant associations (such as education and income) might be due to limited statistical power; larger samples are needed for validation. Future research should also consider unmeasured variables, including mental health, cognitive function, and digital HL.

## 5. Conclusions

In summary, approximately half (54.2%) of the older adults in Northern Thailand participating in this study demonstrated limited HL. Regular exercise, use of a sitting toilet, and wearing properly fitting shoes were associated with HL levels. These findings may inform community health education strategies aimed at enhancing access to and comprehension of health information among older adults. However, given the cross-sectional design, convenience sampling, and regional focus, these results should be interpreted as associations and not causal relationships.

## Figures and Tables

**Table 1:** Sociodemographic characteristics among older adults (n = 177).

Parameters		n (%)
Age (years), mean ± SD		68.3 ± 5.9
Sex	Female	124 (70.1)
	Male	53 (29.9)
Marital status	Single	9 (5.1)
	Married	117 (66.1)
	Divorced/widow	51 (28.8)
Education level	Elementary school or lower	148 (83.6)
	Secondary school	25 (14.1)
	Bachelor’s degree or higher	4 (2.3)
Monthly income	≤150 US Dollars	163 (92.1)
	>150 US Dollars	14 (7.9)
Co-morbidity		93 (52.5)
Number of medication use	None	77 (43.5)
	<4 types	72 (40.7)
	≥4 types	28 (15.8)
Smoking cigarette		12 (6.8)
Alcohol consumption		46 (26)
Regular exercise		69 (39)
Village health volunteer		41 (23.2)
Living with family		147 (83.1)
History of falls in the past 6 months		27 (15.3)
Gait aids use		7 (4)
Vision	Clearly	82 (46.3)
	Clearly with glasses	82 (46.3)
	Not clear and have no glasses	13 (7.4)
Hearing	Clearly	151 (85.3)
	Clearly with hearing aids	3 (1.7)
	Not clear and have no hearing aids	23 (13.0)

**Table 2:** Home environmental factors and fall prevention behaviors among older adults (n = 177).

Parameters	n (%)
*Home Environmental Factors*	
Ground floor bedroom	94 (53.1)
Even-dry surface	162 (91.5)
Handrails on stairs	152 (85.9)
Clear walkway	175 (98.9)
Sufficient lighting	173 (97.7)
Handrails in bathroom	60 (33.9)
Sitting toilet	130 (73.4)
Elevated bed	154 (87)
Countertop kitchen	154 (87)
Even surface around house	159 (89.8)
*Fall Prevention Behaviors*	
Wearing properly fitting shoes	133 (75.1)
Turning-on the light in the bathroom	154 (87)
Wiping water spilled on the floor	173 (97.7)
Sit when taking shower	56 (31.6)
Slowly changing position	134 (75.7)
Wearing appropriately sized clothes	170 (96)
Cleaning up house	172 (97.2)
Using handrails	114 (64.4)
Using walking aids if advised	52 (29.4)
Screening for fall risk annually	89 (50.3)

**Table 3:** Multiple linear regression analysis for investigating factors associated with health literacy scores among older adults.

Factors	n (%)	β ± SE (95%CI)			
		Total Score of HL	Subdomain 1 HL: Healthcare	Subdomain 2 HL: Disease Prevention	Subdomain 3 HL: Health Promotion
*Sociodemographic Factors*					
Monthly income < 150 US Dollars	163 (92.1)	1.07 ± 1.79 (−2.47, 4.61)	3.15 ± 1.91 (−0.61, 6.92)	−1.13 ± 2.21 (−5.49, 3.22)	2.06 ± 2.72 (−3.31, 7.43)
Co-morbidity	93 (52.5)	−0.68 ± 0.99 (−2.64, 1.27)	−0.93 ± 1.05 (−3.00, 1.15)	0.14 ± 1.22 (−2.27, 2.54)	1.28 ± 1.51 (−1.71, 4.27)
Smoking cigarette	12 (6.8)	−2.29 ± 1.91 (−6.07, 1.48)	−1.96 ± 2.03 (−5.968, 2.059)	−2.22 ± 2.35 (−6.86, 2.42)	−1.54 ± 2.77 (−7.01, 3.93)
Regular exercise	69 (39)	2.73 ± 1.019 (0.71, 4.74) **	2.47 ± 1.08 (0.33, 4.61) *	3.18 ± 1.25 (0.71, 5.66) *	1.78 ± 1.56 (−1.30, 4.86)
Living with family	147 (83.1)	−1.84 ± 1.264 (−4.34, 0.65)	−0.03 ± 1.35 (−2.68, 2.63)	−1.52 ± 1.56 (−4.59, 1.55)	−0.91 ± 2.05 (−4.96, 3.13)
Gait aid use	7 (4)	−3.87 ± 2.45 (−8.70, 0.97)	−5.15 ± 2.61 (−10.29, −0.01) *	−3.64 ± 3.01 (−9.59, 2.31)	−7.48 ± 3.54 (−14.47, −0.49) *
*Home Environmental Factors*					
Handrails in the bathroom	60 (33.9)	−0.02 ± 1.08 (−2.16, 2.12)	−0.56 ± 1.15 (−2.83, 1.71)	0.18 ± 1.33 (−2.45, 2.81)	1.20 ± 1.68 (−2.12, 4.51)
Sitting toilet	130 (73.4)	6.38 ± 1.29 (3.83, 8.92) **	6.31 ± 1.37 (3.59, 9.02) **	6.45 ± 1.59 (3.32, 9.59) **	3.18 ± 1.91 (−0.60, 6.95)
Countertop kitchen	154 (87)	1.73 ± 1.58 −1.39, 4.85	2.65 ± 1.68 (−0.68, 5.97)	3.24 ± 1.95 (−0.60, 7.08)	−3.04 ± 2.32 (−7.63, 1.55)
*Fall Prevention Behavior Factors*					
Wearing properly fitting shoes	133 (75.1)	2.54 ± 1.17 (0.22, 4.86) *	1.73 ± 1.25 (−0.74, 4.20)	1.76 ± 1.44 (−1.09, 4.61)	−0.06 ± 1.77 (−3.57, 3.44)
Turning-on the light in the bathroom	154 (87)	1.55 ± 1.50 (−1.40, 4.51)	2.33 ± 1.59 (−0.82, 5.47)	0.67 ± 1.84 (−2.97, 4.30)	−3.13 ± 2.27 (−7.61, 1.34)
Constant		30.98 (20.34, 41.62)	25.61 (14.28, 36.93)	30.10 (13.00, 43.19)	43.19 (27.37, 59.00)
Adjusted R2		0.38	0.30	0.21	0.21

Note. Adjusted with age, sex, and education. Reference groups for the variables are as follows: ‘Monthly income < 150 USD’ is compared with ‘Monthly income ≥ 150 USD’; ‘Co-morbidity’ with ‘No co-morbidity’; ‘Smoking cigarette’ with ‘No smoking’; ‘Regular exercise’ with ‘No regular exercise’; ‘Living with family’ with ‘Not living with family’; and ‘Gait aid use’ with ‘No gait aid use’. Constant = intercept of the regression model, representing the estimated health literacy score when all predictors are set to zero. Abbreviations: β = beta coefficient; SE = standard error; 95% CI = 95% confidence interval. * *p* < 0.05; ** *p* < 0.001.

## Data Availability

The original data supporting the findings of this study are not publicly available due to sensitivity concerns; however, they can be obtained from the corresponding author upon reasonable request. Data are in controlled access data storage at University of Phayao, Thailand.

## Abbreviations

The following abbreviations are used in this manuscript:

HL	Health literacy
CI	Confidence interval
HLS-EU-Q47	The European Health Literacy Survey Questionnaire
ADL	Activities of daily living
WHO	World Health Organization
IOC	Index of item objective congruence
SES	Socioeconomic status
HBM	Health belief model

## Appendix A

**Table A1:** Univariable analysis for investigating factors associated with health literacy scores among older adults.

Parameters		HL Scores (Mean ± SD)	*p*-Value	VIF Value
*Sociodemographic Characteristics*				
Monthly income	≤150 USD/month	34.2 ± 7.5	0.045 *	1.73
	>150 USD/month	38.3 ± 7.0		
Co-morbidity	Yes	33.1 ± 7.5	0.010 *	3.61
	No	36.0 ± 7.3		
Regular exercise	Yes	36.4 ± 7.7	0.006 *	1.34
	No	33.3 ± 7.2		
Smoking cigarette	Yes	30.1 ± 3.4	0.038 *	1.34
	No	34.8 ± 7.6		
Living with family	Yes	37.1 ± 6.2	0.036 *	1.48
	No	33.9 ± 7.7		
Gait aid use	Yes	28.7 ± 3.4	0.036 *	1.23
	No	34.7 ± 7.5		
Gender	Female	34.7 ± 7.5	0.513	1.56
	Male	33.9 ± 7.7		
Marital status	Single	38.3 ± 9.5	0.282	1.65
	Married	34.2 ± 7.5		
	Divorced/widow	34.6 ± 7.0		
Education level	Elementary school or lower	33.9 ± 7.6	0.072	1.73
	Secondary school	36.3 ± 6.3		
	Bachelor’s degree or higher	41.2 ± 7.2		
Number of medication use	None	35.6 ± 7.5	0.077	3.47
	<4 types	33.8 ± 7.6		
	≥4 types	32.5 ± 6.8		
Alcohol consumption	Yes	34.0 ± 7.3	0.158	1.47
	No	35.8 ± 7.9		
Village health volunteer	Yes	36.1 ± 7.0	0.109	1.39
	No	34.0 ± 7.6		
Vision	Clearly	34.1 ± 7.3	0.184	1.38
	Clearly with glasses	35.3 ± 7.8		
	Not clear and have no glasses	31.4 ± 6.7		
Hearing	Clearly	35.0 ± 7.5	0.054	1.37
	Clearly with hearing aids	36.3 ± 5.8		
	Not clear and have no hearing aids	31.0 ± 7.5		
History of falls in the past 6 months	Yes	33.7 ± 7.6	0.559	1.47
	No	34.6 ± 7.5		
*Home Environmental Factors*				
Handrails in the bathroom	Yes	36.6 ± 6.8	0.006 *	1.54
	No	33.4 ± 7.6		
Sitting toilet	Yes	36.7 ± 6.8	<0.001 **	1.91
	No	28.4 ± 5.6		
Countertop kitchen	Yes	35.4 + 7.4	<0.001 **	1.63
	No	28.6 ± 5.8		
Ground floor bedroom	Yes	35.0 ± 7.0	0.375	1.30
	No	33.9 ± 8.0		
Even-dry floor	Yes	34.4 ± 7.7	0.691	1.44
	No	35.2 ± 4.5		
Handrails on stairs	Yes	34.4 ± 7.8	0.880	1.54
	No	34.7 ± 5.9		
Clear walkway	Yes	37.6 ± 5.0	0.559	1.24
	No	34.4 ± 7.5		
Sufficient lighting	Yes	35.6 ± 3.0	0.775	1.41
	No	34.5 ± 7.6		
Platform bed	Yes	34.8 ± 7.6	0.128	1.28
	No	32.3 ± 6.6		
Even surface around house	Yes	34.4 ± 7.7	0.635	1.30
	No	35.2 ± 5.2		
*Fall Prevention Behavior Factors*				
Wearing properly fitting shoes	Yes	35.8 ± 7.6	<0.001 **	1.48
	No	30.5 ± 5.9		
Turning-on the light in the bathroom	Yes	35.1 ± 7.2	0.004 *	1.38
	No	30.4 ± 8.3		
Wiping water spilled on the floor	Yes	34.5 ± 7.6	0.537	1.24
	No	32.2 ± 4.9		
Sit when taking shower	Yes	35.3 ± 7.4	0.315	1.54
	No	34.1 ± 7.4		
Slowly changing position	Yes	34.1 ± 7.7	0.191	1.52
	No	35.7 ± 6.9		
Wearing appropriately sized cloths	Yes	34.5 ± 7.6	0.785	1.34
	No	35.4 ± 4.5		
Cleaning up house	Yes	34.5 ± 7.5	0.778	1.27
	No	33.5 ± 7.4		
Using handrails	Yes	35.0 ± 8.1	0.261	1.59
	No	33.6 ± 6.4		
Using a walking aid if advised	Yes	34.3 ± 8.5	0.848	1.31
	No	34.6 ± 7.1		
Screening for fall risk annually	Yes	35.1 ± 7.0	0.272	1.38
	No	33.9 ± 8.0		

*Note. HL* Health literacy, *VIF* Variance inflation factor. The categorical data are compared using the Chi square test for each comparison. * *p* value < 0.05, ** *p* value < 0.001.
